# Injectable Alginate Complex Hydrogel Loaded with Dual-Drug Nanovectors Offers Effective Photochemotherapy against Triple-Negative Breast Cancer

**DOI:** 10.1021/acs.biomac.3c01426

**Published:** 2024-02-21

**Authors:** Yu-Hsiang Lee, Chih-Ting Lin

**Affiliations:** † Department of Biomedical Sciences and Engineering, National Central University, Taoyuan City 320317, Taiwan R.O.C; ‡ Department of Chemical and Materials Engineering, National Central University, Taoyuan City 320317, Taiwan R.O.C

## Abstract

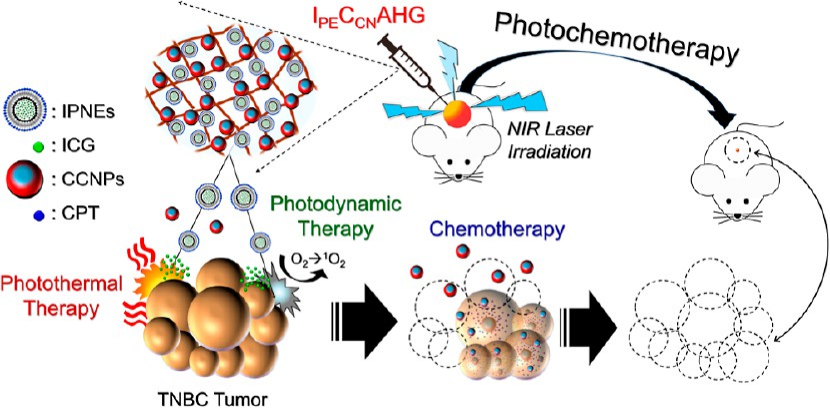

Triple-negative breast cancer (TNBC), accounting for approximately 20% of breast cancer cases, is a particular subtype that lacks tumor-specific targets and is difficult to treat due to its high aggressiveness and poor prognosis. Chemotherapy remains the major systemic treatment for TNBC. However, its applicability and efficacy in the clinic are usually concerning due to a lack of targeting, adverse side effects, and occurrence of multidrug resistance, suggesting that the development of effective therapeutics is still highly demanded nowadays. In this study, an injectable alginate complex hydrogel loaded with indocyanine green (ICG)-entrapped perfluorocarbon nanoemulsions (IPNEs) and camptothecin (CPT)-doped chitosan nanoparticles (CCNPs), named I_PE_C_CN_AHG, was developed for photochemotherapy against TNBC. IPNEs with perfluorocarbon can induce hyperthermia and generate more singlet oxygen than an equal dose of free ICG upon near-infrared (NIR) irradiation to achieve photothermal and photodynamic therapy. CCNPs with positive charge may facilitate cellular internalization and provide sustained release of CPT to carry out chemotherapy. Both nanovectors can stabilize agents in the same hydrogel system without interactions. I_PE_C_CN_AHG integrating IPNEs and CCNPs enables stage-wise combinational therapeutics that may overcome the issues described above. With 60 s of NIR irradiation, I_PE_C_CN_AHG significantly inhibited the growth of MDA-MB-231 tumors in the mice without systemic toxicity within the 21 day treatment. We speculate that such anticancer efficacy was accomplished by phototherapy followed by chemotherapy, where cancer cells were first destroyed by IPNE-derived hyperthermia and singlet oxygen, followed by sustained damage with CPT after internalization of CCNPs; a two-stage tumoricidal process. Taken together, the developed I_PE_C_CN_AHG is anticipated to be a feasible tool for TNBC treatment in the clinic.

## 1 Introduction

Triple-negative breast cancer (TNBC), which accounts for approximately 20% of breast cancer cases, is a particular subtype that lacks the expression of human epidermal growth factor receptor 2 (HER2), estrogen receptor (ER), or progesterone receptor (PR).^1^ In general, TNBC is biologically aggressive since it is highly proliferative and is the subtype with the poorest prognosis due to its highly malignant nature.^2^ Although the development of alternative therapeutics has continued over the last decades, chemotherapy remains the major approach for systemic treatment of TNBC nowadays. However, its applicability and efficacy for clinical use are usually concerning due to the lack of targeting, adverse side effects, and occurrence of multidrug resistance.^2^

Utilization of localized drug delivery systems directly performed in cancerous regions has long been recognized as a promising strategy to reduce side effects caused by off-target drugs. Hydrogels are one of the most commonly used materials since they are able to absorb large amounts of biological fluids and have mechanical properties similar to those of the natural extracellular matrix. Among various biopolymers used for hydrogel preparation, alginate is a linear anionic biomacromolecule which can form a hydrogel through cross-linking with divalent cations such as Mg^2+^, Ca^2+^, and Cu^2+^.^3−5^ Because of their rigid structure and biocompatible properties, alginate-derived hydrogels have been widely exploited in a variety of fields such as drug delivery, tissue engineering, and cell culture.^6−8^

Joint therapy through coadministration of anticancer agents and/or methods is a feasible way to reduce multidrug resistance since the effective dosage and/or accumulation of each chemotherapeutic in the tumor can be diminished, through which the issue of chemoresistance can be resolved accordingly.^9^ Among a variety of chemotherapeutic adjuvants, near-infrared (NIR)-mediated phototherapy has gained increasing attention owing to its several merits including noninvasiveness, superior tissue penetration efficiency, and capability to aid subsequent drug transport and/or absorption.^10,11^ In general, phototherapy functions through hyperthermia or free radicals generated by photosensitizers under light exposure. The former can cause irreversible cell damage with temperatures >45 °C called photothermal therapy (PTT), whereas the latter is able to disturb cell physiology and thus induce cell death named photodynamic therapy (PDT).^12,13^

Indocyanine green (ICG) is a USFDA-approved fluorophore and has been extensively utilized as a photosensitizer for cancer phototherapy, including for colorectal, skin, and breast cancer treatments^14−16^ because it is able to generate both singlet oxygen and hyperthermia upon NIR irradiation to perform PDT and PTT, respectively. However, several disadvantages, such as photo- and thermal susceptibility, rapid clearance in circulation, and concentration-dependent agglomeration,^17,18^ severely hamper the applicability of ICG in clinical practice. In addition, it is critical to deliver all the anticancer agents (e.g., photosensitizers and chemo-drugs) simultaneously to tumor sites without interactions during transportation.

Hydrogels in association with drug nanovectors may provide a feasible means to handle multiple agents such as ICG and anticancer drugs in the same delivery system without the aforementioned concerns since nanoencapsulation can offer enhanced stability, integrity, and safety to payloads.^19^ In this study, we sought to fabricate an injectable alginate composite hydrogel loaded with ICG-entrapped perfluorocarbon (PFC) nanoemulsions (IPNEs) and camptothecin (CPT)-doped chitosan nanoparticles (CCNPs), named I_PE_C_CN_AHG, and explore its potential for use in photochemotherapy of TNBC. PFCs are fluorine-substituted anthropogenic hydrocarbons and have long been utilized as oxygen carriers owing to their high oxygen dissolubility compared to water.^20^ Chitosan was selected due to its nature of positive charge that is favorable for cell internalization of CCNPs.^21−23^ Taken together, we anticipate that I_PE_C_CN_AHG could proactively bring oxygen to tumors, which is often hypoxia to promote PDT, provide sustained CPT-derived chemotherapy through the delivery of CCNPs, and consequently achieve successful photochemotherapy of TNBC.

## 2 Materials and Methods

### 2.1 Preparation and Evaluation of IPNEs and CCNPs

IPNEs were fabricated using a modified dual emulsification procedure reported previously.^14^ Briefly, a total of 1 mg of ICG (Sigma-Aldrich, St. Louis, MO, USA) dissolved in 1 mL of methanol [50% (v/v)] was first added to 1.8 mL of perfluorooctyl bromide (PFOB, Sigma-Aldrich) containing polyethoxylated fluorosurfactant (2 wt %), and the mixture was processed by sonication for 5 min. The produced green-milky emulsions were swiftly added to 10 mL of Pluronic F68 (PF68, Sigma-Aldrich) solution (5 wt %) and subjected to the second sonication for 10 min, whereby the IPNEs formed with W/PFC/W configuration were obtained. After being washed twice with deionized (DI) water, the IPNEs were stored in the dark at 4 °C.

CCNPs were synthesized by an ionic gelation method reported previously.^24^ In brief, chitosan (100–300 kDa, Thermo Fisher Scientific, Waltham, MA, USA) and sodium tripolyphosphate (STPP) were first dissolved in acetic acid (0.3% w/v) and DI water (0.1% w/v), respectively. Afterward, 5 mL of chitosan solution containing 600 μg of CPT (Sigma-Aldrich) was mixed with magnetic stirring at a speed of 2000 rpm. Then the STPP solution was added dropwise to the above mixture, and the agitation lasted for 3 h to obtain the CCNPs. After washing twice with DI water, the synthesized CCNPs were stored at 4 °C until use.

Both the size distribution and zeta potential of the IPNEs and CCNPs were evaluated by dynamic light scattering (DLS, NanoBrook 90plus, Brookhaven Instruments, Holtsville, NY) set by 15 and 90° of measurement angles and analyzed using the Particle Solutions software (Brookhaven Instruments). After lyophilization followed by surface coating with gold, the morphology of each type of nanocarrier was observed by using scanning electron microscopy (SEM, HITACHI SU8200, HITACHI, Tokyo, Japan) with a 10 kV accelerating voltage. The encapsulation and loading ratios of ICG and CPT in each nanovector were assessed by spectrophotometry reported previously.^16^

### 2.2 Fabrication and Characterization of I_PE_C_CN_AHG

**Figure 1:**
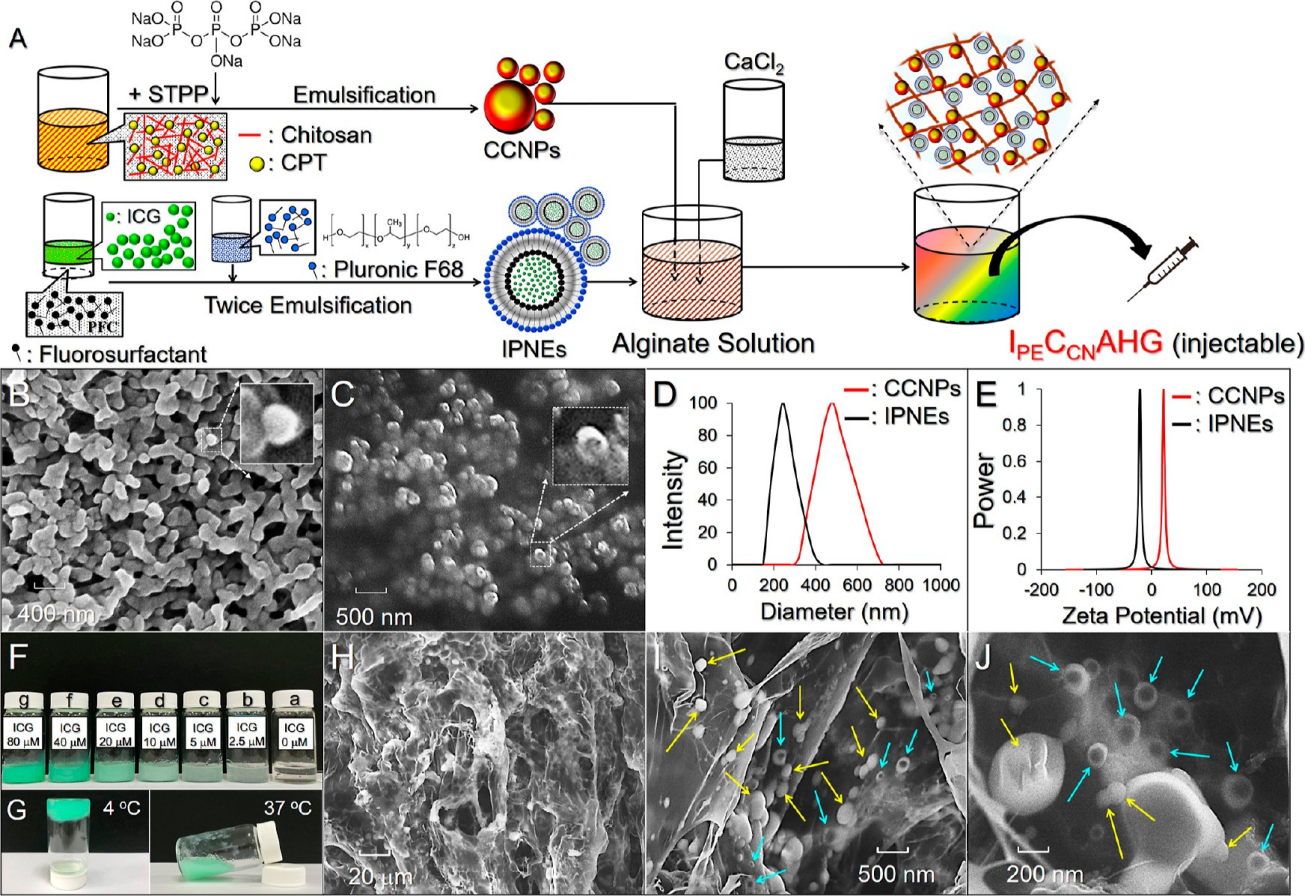
Preparation and characterization of CCNPs, IPNEs, and I_PE_C_CN_AHG. (A) Schematic diagram showing the fabrications of CCNPs, IPNEs, and I_PE_C_CN_AHG. (B,C) SEM images of CCNPs (B) and IPNEs (C) at 18,000× magnification. (D,E) Size distribution (D) and zeta potential (E) of the CCNPs and IPNEs detected by DLS. (F) Appearances of I_PE_C_CN_AHG with different ICG doses. Sample #a is a blank AHG. The concentrations of CPT in samples #b–#g are equally set as 10 μM. (G) Photographs of I_PE_C_CN_AHG at 4 and 37 °C. (H–J) SEM images of I_PE_C_CN_AHG taken at 500× (H), 8,000× (I), and 20,000× (J) magnification. Yellow and blue arrows in parts (I,J) indicate CCNPs and IPNEs, respectively.

Sodium alginate (viscosity: 100–300 mPa’s, DuPont, Wilmington, DE, USA) was first dissolved in DI water (1.5% w/v) at ambient temperature by magnetic stirring until a clear solution was obtained. Next, a calcium chloride solution (0.1%, w/v) containing designated amounts of the IPNEs and CCNPs was added to the above alginate solution under 800 rpm agitation. The mixture was continuously stirred at 800 rpm under ambient temperature for 20 min, after which the I_PE_C_CN_AHG was obtained. The I_PE_C_CN_AHG was stored in the dark at 4 °C until use. The structure, configuration, and nanovector distribution of I_PE_C_CN_AHG were detected by SEM. The fabrication of I_PE_C_CN_AHG including the preparation of IPNEs and CCNPs is schematically presented in Figure 1A.

### 2.3 Assessment of the Stability and Drug Release Efficiency of I_PE_C_CN_AHG

The in vitro stability of the ICG encapsulated in IPNEs and I_PE_C_CN_AHG, as well as the drug release kinetics of the CPT entrapped in CCNPs and I_PE_C_CN_AHG, was analyzed by a spectrophotometric approach. Both the IPNEs and I_PE_C_CN_AHG with 40 μM ICG were incubated at 4 and 37 °C, in which the IPNEs were homogeneously distributed in 5 mL of PBS, whereas the I_PE_C_CN_AHG with 1.5 mL was placed in a quartz cuvette. After 3, 6, 12, 24, and 48 h, the IPNEs were collected by centrifugation, while the I_PE_C_CN_AHG was steadily maintained in the cuvette, and they were subjected to spectrophotometry at λ = 780 nm to measure the quantity of ICG remaining in each material.

To assess the release kinetics of CPT, CCNPs and I_PE_C_CN_AHG with equal 300 μM CPT were separately placed in 10 mL of PBS. After incubation at 4 or 37 °C for 3, 6, 12, 24, and 48 h, the supernatant of each group was subjected to spectrophotometry at λ = 370 nm to measure the quantity of CPT released.

To evaluate how NIR irradiation affects the drug release efficiency of the composite hydrogel, I_PE_C_CN_AHGs with 300 μM CPT were exposed to NIR and the supernatants collected after 1, 2, 3, 4, and 5 min of NIR irradiation were subjected to spectrophotometry at λ = 370 nm. The temperature of the hydrogel system under NIR irradiation was simultaneously detected using a digital thermometer every 60 s for 5 min. NIR irradiation was conducted using an 808 nm laser with an output intensity of 6 W/cm^2^.

### 2.4 Evaluation of I_PE_C_CN_AHG-Induced Hyperthermia Effects

A 100 μL aliquot of I_PE_C_CN_AHGs containing 2.5, 5, 10, 20, 40, 80, or 160 μM ICG, where the CPT concentration was fixed at 50 μM for each group, was separately placed in 100 μL of DI water. The temperature of each group was detected using a digital thermometer every 30 s for 5 min during NIR exposure (808 nm; 6 W/cm^2^).

### 2.5 Evaluation of I_PE_C_CN_AHG-Induced Singlet Oxygen Production

The quantity of singlet oxygen induced by I_PE_C_CN_AHG under NIR exposure (808 nm; 6 W/cm^2^) was measured using a commercial singlet oxygen sensor green (SOSG) detection kit (Life Technologies, Carlsbad, CA, USA) according to the manufacturer’s instructions. The concentrations of ICG used in I_PE_C_CN_AHG were set to 2.5, 5, 10, 20, 40, 80, and 160 μM, where the CPT concentration was fixed at 50 μM for all groups. The level of SOSG-induced fluorescence was measured by spectrofluorometry (excitation/emission wavelength = 488/525 nm) every 60 s for 5 min and was quantified by relative fluorescence units (RFUs).

### 2.6 Evaluation of the Rheological and Thermal Properties of I_PE_C_CN_AHG

The rheological properties of I_PE_C_CN_AHG with different ICG/CPT dose ratios (40/20 and 80/40 μM) were measured using a rheometer (Discovery HR-1, TA Instruments, New Castle, DE, USA) in association with a temperature controller. The storage modulus (*G*′), loss modulus (*G*″), and complex viscosity (η*) vs angular frequency (rad/s) were measured in oscillatory mode at 37 °C for each sample.

I_PE_C_CN_AHG with [ICG]/[CPT] = 80/40 μM was subjected to thermogravimetric analysis (TGA) in association with derivative thermogravimetry (DTG) analyses (PYRIS 1, PerkinElmer, Waltham, MA, USA) after lyophilization. The sample was heated from 50 to 900 °C at an increasing rate of 10 °C/min under a nitrogen environment. A blank alginate hydrogel (AHG) was employed as the control group in both the rheological and thermal analyses.

### 2.7 Assessment of Degradation of I_PE_C_CN_AHG In Vitro

The in vitro degradation of I_PE_C_CN_AHG was evaluated based on the dry weight loss under body temperature over time. Briefly, 7 mL of I_PE_C_CN_AHG containing 40/20 μM ICG/CPT was aliquoted into seven tubes of PBS [hydrogel/PBS = 1:1 (v/v)], and all groups were maintained at 37 °C in the dark. One tube of I_PE_C_CN_AHG was lyophilized and weighed every 24 h for 7 days. The degradation ratio (*R*_d_) was calculated by *R*_d_ = 1 – (*W*_t_/*W*_0_), where *W*_0_ and *W*_t_ represent the dry weight of the I_PE_C_CN_AHG used before heating and measured at a specific time *t* > 0, respectively.

### 2.8 Cell Culture

Human breast adenocarcinoma MDA-MB-231 cells (ATCC HTB-26, TNBC) were cultured in Dulbecco’s modified Eagle medium supplemented with 10% fetal bovine serum and 100 U/mL penicillin/streptomycin at 37 °C with 5% CO_2_.

### 2.9 Cytotoxicity of I_PE_C_CN_AHG In Vitro

The phototherapeutic effect of photosensitizer-containing AHG on TNBC cells was first examined using IPNEs-loaded AHG (I_PE_AHG) as the test material. After incubation at 37 °C for 24 h, 1 × 10^5^ MDA-MB-231 cells per well in 96-well culture plates were separately treated with none (blank), NIR, AHG + NIR, free ICG + NIR, and I_PE_AHG + NIR, in which the ICG doses (if available) were set as 0 (blank gel), 10, 40, 80, or 160 μM. NIR exposure was operated by using an 808 nm laser with an output intensity of 6 W/cm^2^ for 5 min. The cells in all groups were subjected to viability analysis by the MTT assay after incubation at 37 °C for 24 h.

The photochemotherapeutic effect of I_PE_C_CN_AHG on TNBC cells was further examined as the dose of IPNEs was determined based on the results of I_PE_AHG-mediated anticancer assays described above. 1 ×10^5^ MDA-MB-231 cells per well in 96-well culture plates were separately treated with none (blank), NIR, free CPT, I_PE_C_CN_AHG, or I_PE_C_CN_AHG + NIR, in which the CPT doses (if available) were set as 0 (i.e., I_PE_AHG), 50, 150, 300, or 600 μM. All groups were subjected to viability analyses by calcein-AM staining and MTT assays after incubation at 37 °C for 24 h.

### 2.10 Animal Model

All animal experiments were operated in accordance with the guidelines approved by the Institutional Animal Care and Use Committee at Cathay General Hospital (Taiwan ROC, approval number: CGH-IACUC-112–002). Implantation of the TNBC tumor in vivo was performed by subcutaneously injecting 1 × 10^7^ MDA-MB-231 cells into the flank region of each BALB/c nude mouse (age: 7–8 weeks, weight: 25–30 g) purchased from BioLASCO (Taipei, Taiwan ROC). Tumor size (*V*) was monitored and estimated every 48 h using the equation *V* = (*L* × *W*^2^)/2 where *L* and *W* denote the tumor length in the major and minor axes, respectively. The animal study was initiated when the tumor size reached 80–100 mm^3^.

### 2.11 In Vivo Anticancer Studies

Tumor-bearing mice were randomly divided into five groups: (1) PBS, (2) free CPT, (3) free ICG + NIR, (4) I_PE_C_CN_AHG, and (5) I_PE_C_CN_AHG + NIR (4 mice for each group), where all kinds of agents in 80 μL were applied to tumors intratumorally every 3 days for total 21 days. NIR irradiation was operated using an 808 nm laser with an intensity of 6 W/cm^2^ for 1 min. The doses of ICG and/or CPT in all groups (if available) were set to be the same as that provided by the I_PE_C_CN_AHG and those were decided based on the in vitro cytotoxicity results.

The body weights, appearances of the tumor site, and tumor sizes of all of the experimental mice were recorded every 72 h before the next treatment throughout the time course. All tumor-bearing mice were sacrificed on the 21st day or when the tumor size was larger than 2000 mm^3^ according to the animal protocol. Tumors and five organs, including the kidney, lung, spleen, liver, and heart, were excised from all of the experimental mice immediately after sacrifice for the subsequent analysis.

### 2.12 Histological Studies

All tissue specimens were prepared using a routine histological process including ethanol dehydration, formalin fixation, xylene clearance, and paraffin embedment as described elsewhere.^25^ Organ tissues were stained with hematoxylin and eosin (H&E), while the tumors were subjected to *K*_i_-67 and caspase-3 immunohistochemical (IHC) assays. All tissue microphotographs were analyzed using Motic DSA software (Motic, Kowloon, Hong Kong). The expression levels of *K*_i_-67 and caspase-3 in all tumors were quantitatively analyzed using ImageJ.

### 2.13 In Vivo Biocompatibility Analyses

The blood of all of the experimental mice was collected 24 h before treatment (day-1) and right before sacrifice. All blood samples were immediately subjected to biochemical analyses, where the number of blood cells and expression levels of liver and kidney serum markers were detected using a blood analyzer (FUJI DRI-CHEM 4000i, FUJIFILM, Tokyo, Japan). For the groups with PBS, free CPT, I_PE_C_CN_AHG, and I_PE_C_CN_AHG + NIR, the quantity of CPT in their five organs and tumors was analyzed by spectrophotometry at λ = 370 nm after sacrifice.

### 2.14 Statistical Analysis

All data are the results of ≥ three independent experiments and are presented as the mean ± standard deviation (SD). Statistical analysis was performed using MedCalc software. Comparisons were calculated using Student’s *t*-test followed by Dunnett’s posthoc test. Statistical significance was accepted at *P* < 0.05 throughout the study.

## 3 Results and Discussion

### 3.1 Characterization of IPNEs, CCNPs, and I_PE_C_CN_AHG

Figure 1B,C shows the SEM images of CCNPs and IPNEs, respectively, where the CCNPs are solid nanospheres with rough surfaces (Figure 1B, inset image), while the IPNEs appear as a double-layered structure according to their core–shell configuration (Figure 1C, inset image). All the characteristics of the CCNPs and IPNEs including size, surface charge, encapsulation efficiency of payloads, and drug loading ratio are presented in Table 1.

**Table 1:** Characteristics of CCNPs and IPNSs

	size (nm)	zeta potential (mV)	encapsulation ratio (%)	loading ratio (%, w/w)
CCNPs	483.23 ± 56.6	28.51 ± 3.35	92.6 ± 1.4 (CPT)	0.073 ± 0.012 (CPT)
IPNEs	219.76 ± 13.1	–33.27 ± 4.9	96.5 ± 2.9 (ICG)	0.038 ± 0.006 (ICG)

I_PE_C_CN_AHG is a green semitransparent injectable hydrogel in which the color brightness is positively correlated with the concentration of the involved ICG (Figure 1F). I_PE_C_CN_AHG is thermoresponsive since it has a higher fluidity (i.e., lower viscosity) at 37 °C (Figure 1G/right) than at 4 °C (Figure 1G/left). I_PE_C_CN_AHG was formed with a porous structure as presented in Figure 1H, where quite a few nanoparticles (CCNPs and IPNEs) were embedded in the gel matrix and/or adhered to the fibrous surface (Figure 1I,J), resulting in a hybrid composition for I_PE_C_CN_AHG.

### 3.2 Thermal Stability of I_PE_C_CN_AHG-Encapsulated ICG

**Figure 2:**
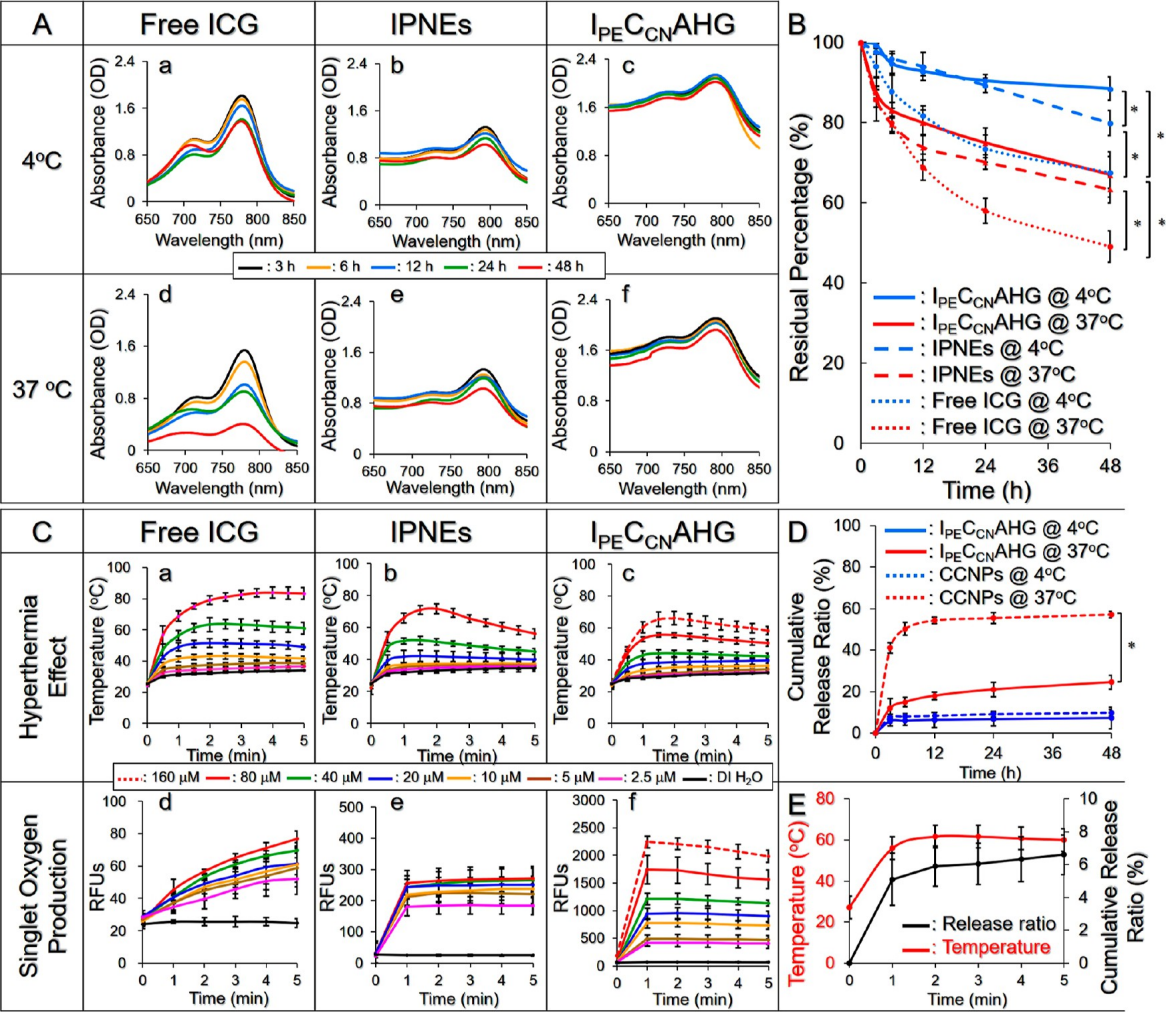
Thermal stability and functionality of I_PE_C_CN_AHG in vitro. (A) UV–vis spectra showing the degradation of ICG in aqueous solution (a,d), IPNEs (b,e), and I_PE_C_CN_AHG (c,f) at 4 (a–c) or 37 °C (d–f) within 48 h. (B) Quantitative analyses of the ICG remaining in DI water, IPNEs, or I_PE_C_CN_AHG under incubation at 4 or 37 °C for 48 h. (C) Hyperthermia effects (a–c) and production of singlet oxygen (d–f) generated by different concentrations of free ICG, IPNEs, and I_PE_C_CN_AHG within 5 min of NIR exposure. (D) CPT release profiles of CCNPs or I_PE_C_CN_AHG under incubation at 4 or 37 °C for 48 h. (E) CPT release profile of I_PE_C_CN_AHG under NIR irradiation for 5 min (black curve). The red curve represents the temperature change of the system within 5 min of NIR irradiation. Values in (B–E) are the mean ± SD (*n* = 3). *P < 0.05.

Figure 2A shows the changes in the spectrophotometric profile of ICG with different formats at 4 (Figure 2A, a–c) or 37 °C (Figure 2A, d–f) within 48 h. ICG in aqueous solution exhibited remarkable degradation compared with that in IPNEs or I_PE_C_CN_AHG under equal temperature settings. Based on the absorbance analysis, I_PE_C_CN_AHG showed the highest ICG stability among the three settings that approximately 90 and 70% of the encapsulated ICG could be preserved after incubation at 4 and 37 °C, respectively, for 48 h (Figure 2B). These results clearly demonstrate that I_PE_C_CN_AHG was able to significantly enhance the thermal stability of the encapsulated ICG, and we reason that such efficacy was attributed to the double protection provided by the PFC nanoemulsions and AHG.

### 3.3 Effects of Hyperthermia and Singlet Oxygen Production of I_PE_C_CN_AHG

Figure 2C/a–c shows the hyperthermia effects generated by ICG in different formats and at different concentrations within 5 min of NIR irradiation. All three settings exhibited similar temperature change patterns and enabled an ICG dose-dependent hyperthermia effect upon NIR irradiation. However, one may notice that the hyperthermia generated by the encapsulated ICG was lower than that generated by free ICG under the same dose setting. Such outcomes could be explained by the fact that the hyperthermia produced by IPNEs or I_PE_C_CN_AHG was merely caused by partially released ICG that was different from ICG solution where all the ICG molecules can simultaneously react with NIR. Moreover, demulsification and sol–gel transformation toward increased fluidity occurring under NIR exposure are heat absorption processes^26,27^ that may deprive the thermal energy given to the system. Therefore, the level of temperature increase resulting from I_PE_C_CN_AHG was milder than that led by IPNEs and/or free ICG. Nevertheless, these results demonstrate that I_PE_C_CN_AHG is definitely able to provide effective PTT (*T* ≥ 45 °C) as the dose of ICG entrapped in the IPNEs is set to ≥80 μM.

Similarly, all three groups can produce singlet oxygen in a dose-dependent manner upon NIR irradiation (Figure 2C/d–f), and their production can be ordered by I_PE_C_CN_AHG ≫ IPNEs > free ICG throughout the dose range that is opposite to the rank of hyperthermia degree. Based on RFU analysis, the I_PE_C_CN_AHG with 80 μM ICG was able to produce 20.4- and 7.1-fold higher amounts of singlet oxygen compared to that generated by equal doses of free ICG and IPNEs, respectively, after 5 min of NIR irradiation. We reason that such enhanced singlet oxygen production by I_PE_C_CN_AHG was attributed to (1) incorporation of IPNEs in which the constituent PFOB possesses excellent oxygen solubility [527 mL(O_2_)/L_PFOB_]^28^ and (2) vigorous stirring during hydrogel fabrication by which oxygen at the gas–liquid interface can be brought into the gel.

### 3.4 Drug Release Kinetics of CPT with and without NIR Irradiation

Figure 2D shows the drug release profiles of CPT from CCNPs or I_PE_C_CN_AHG at 4 and 37 °C within 48 h. All groups expressed a biphasic release profile consisting of quick release in the first 3 h followed by slow sustained release thereafter. Both systems exhibited *a* < 10% CPT release rate (*P* = NS) at 4 °C, while 57.2 and 24.6% were obtained for the CCNPs and I_PE_C_CN_AHG, respectively, after incubation at 37 °C for 48 h. These results show that the release efficiency of CPT from I_PE_C_CN_AHG is susceptible to environmental temperature and is advantageous for use in vivo.

To understand the drug release behavior of I_PE_C_CN_AHG during phototherapy, we examined the efficiency of CPT release from I_PE_C_CN_AHG under NIR exposure. As shown in Figure 2E, the encapsulated CPT was found to be continuously released under NIR exposure, and a release ratio of 6.61 ± 1.21% was achieved after 5 min. Considering that the systemic temperature was maintained at ∼62 °C after 1 min of NIR irradiation (Figure 2E) which is about the glass-transition temperature (*T*_g_) of sodium alginate (∼64 °C)^29^ but much lower than the *T*_g_ of chitosan (∼150 °C),^30^ we speculate that upon NIR irradiation, the matrix of the AHG, but not CCNPs, rapidly disintegrated. Therefore, CPT was still retained in the CCNPs and gave a moderate drug release efficiency (<10%) consequently. In contrast to the confined release efficiency under static conditions, as shown in Figure 2D, our data demonstrate that the release ratio of CPT from I_PE_C_CN_AHG can be greatly enhanced by NIR irradiation. Furthermore, these results imply that chemotherapy will be predominantly carried out through the delivery of CCNPs rather than free CPT molecules to cells after phototherapy.

### 3.5 Mechanical and Thermal Properties of I_PE_C_CN_AHG

**Figure 3:**
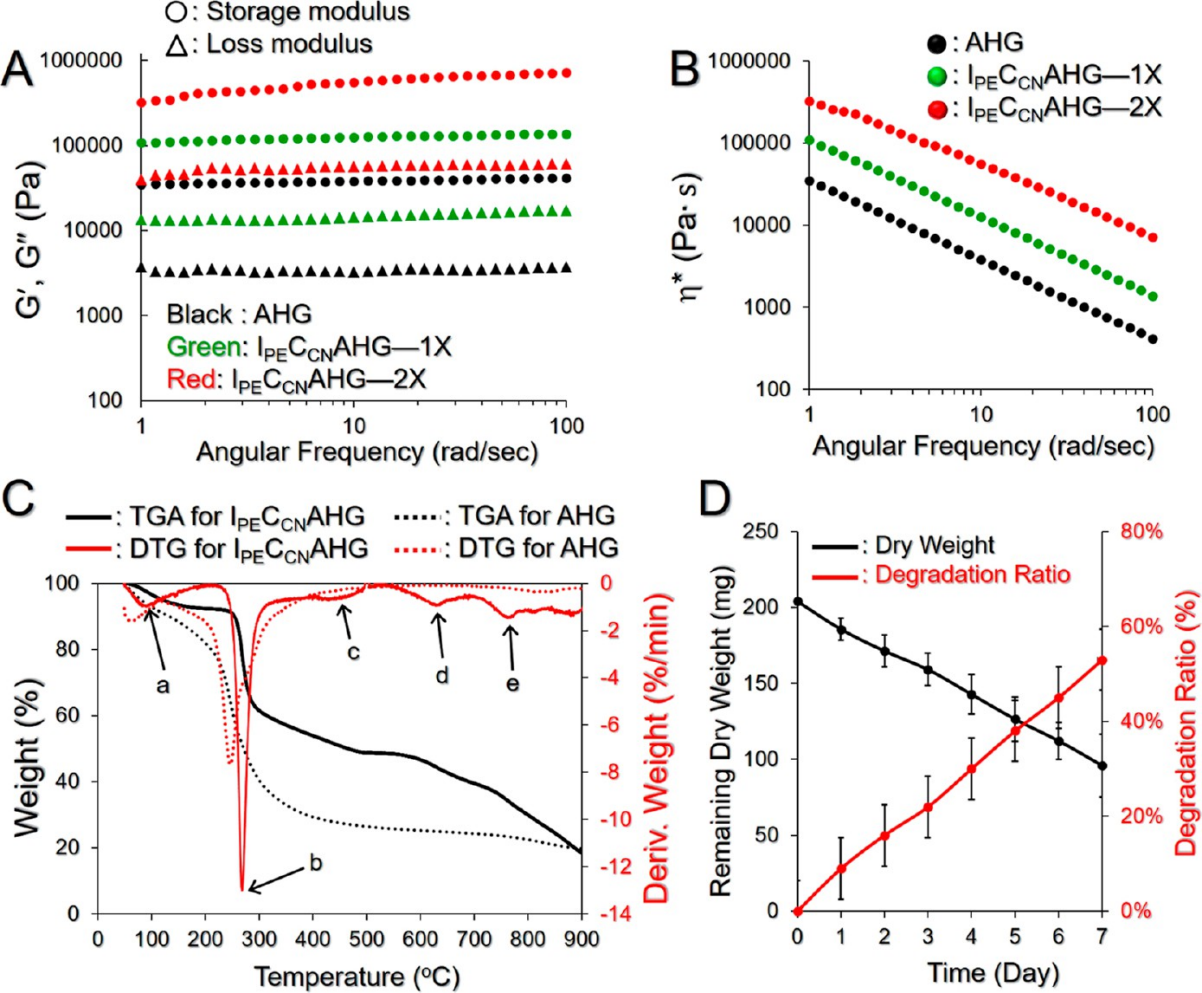
Rheological and thermal properties of I_PE_C_CN_AHG in vitro. (A,B) Analyses of the storage modulus (*G*′; A), loss modulus (*G*″; A), and complex viscosity (η*; B) vs angular frequency of the I_PE_C_CN_AHG loaded with different amounts of nanovectors. 1X and 2X denote that the [ICG]/[CPT] in the I_PE_C_CN_AHG was set as 40/20 and 80/40 μM, respectively. (C) TGA and DTG profiles of the I_PE_C_CN_AHG and AHG. Points a, b, c, d, and e denote the five weight-loss peaks on the DTG curve of I_PE_C_CN_AHG. (D) Amount of remaining dry weight and degradation profile of I_PE_C_CN_AHG under incubation in PBS at 37 °C for 7 days. Values are the mean ± SD (*n* = 3).

Figure 3A shows the rheological behavior of I_PE_C_CN_AHG under rotating impacts at 37 °C. Our data show that the incorporation of nanovectors (CCNPs and/or IPNEs) did not change the phase of the hybrid hydrogel since I_PE_C_CN_AHG was able to maintain a steady gelatinous state under *a* ≤ 100 rad/s of angular frequency. However, the doped IPNEs and CCNPs may increase the viscosity of I_PE_C_CN_AHG (Figure 3B) so that both elasticity and fluidity decrease with increasing number of nanovectors embedded in the hydrogel. Such results could possibly be attributed to the incorporation of nanovectors because they may decrease the porosity of the hydrogel and/or interfere with the fibrous interconnections since the IPNEs and CCNPs tightly coalesced with the fiber networks as shown in Figure 1H.

Figure 3C shows the TGA and DTG profiles of I_PE_C_CN_AHG under heating from 50 to 900 °C, where a five-stage thermal degradation can be detected as indicated by the five weight-loss peaks in the DTG curve (Figure 3C, points a–e). The first stage of degradation with ∼6% weight loss occurring between 80 and 150 °C was likely attributed to losses of adsorbed water and PFOB (*T*_b_ ∼ 142 °C). The second stage appearing at 220–310 °C with ∼35% weight loss was highly correlated with elimination of hydroxyl groups and degradation of the alginate and chitosan backbones.^29,31,32^ The third stage with ∼15% of weight loss at 410–500 °C likely resulted from the decomposition of PF68 and partial disintegration of sodium alginate (*T*_b_ ∼ 495.2 °C).^29,33^ The fourth stage with ∼12% weight loss at 550–680 °C was likely attributed to further degradation of the remaining chitosan, PF68, and sodium alginate. The last stage of I_PE_C_CN_AHG degradation with approximately 14% weight loss at 720–800 °C resulted from decarboxylation and formation of calcium oxide and calcium hydroxide as reported previously.^31^

### 3.6 Degradation of I_PE_C_CN_AHG In Vitro

The degradation of I_PE_C_CN_AHG was assessed by monitoring its dry weight change in 37 °C PBS over time. As shown in Figure 3D, approximately 53% of the weight of I_PE_C_CN_AHG was lost at 37 °C within 7 days. Since I_PE_C_CN_AHG is an ionically cross-linked hydrogel, we reason that such degradation likely occurred due to the release of divalent ions (i.e., Ca^2+^) into the surrounding medium, which was driven by exchange reactions with monovalent cations as reported previously.^34^ Such a biodegradable property of I_PE_C_CN_AHG is anticipated to be favorable for the release of CCNPs which are not discharged during NIR irradiation.

### 3.7 Cytotoxicity of I_PE_C_CN_AHG In Vitro

**Figure 4:**
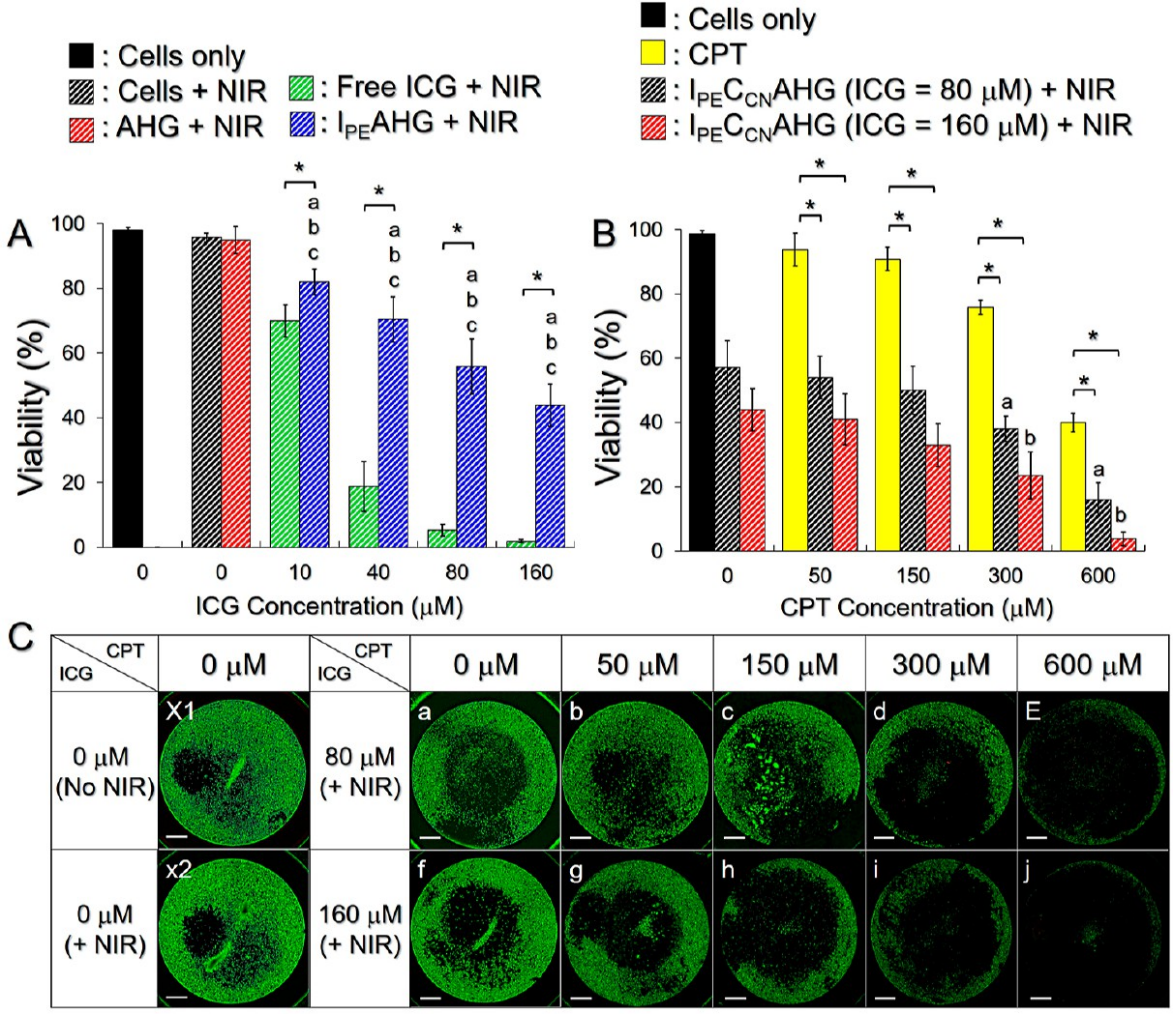
Cytotoxicity of I_PE_C_CN_AHG to TNBC cells in vitro. (A) Viabilities of MDA-MB-231 cells 24 h after various treatments. Values are the mean ± SD (*n* = 3). a, b, and c denote *P* < 0.05 compared to the group with none (cells only), NIR, and AHG + NIR, respectively. *P < 0.05. (B) Viabilities of the MDA-MB-231 cells after treatment with free CPT or I_PE_C_CN_AHG containing different doses of CCNP for 24 h, in which the ICG concentration in the I_PE_C_CN_AHG was fixed at 80 or 160 μM. Values are the mean ± SD (*n* = 3). a and b denote *P* < 0.05 compared to the group with I_PE_C_CN_AHG + NIR where [ICG]/[CPT] = 80/0 μM and 160/0 μM, respectively. *P < 0.05. (C) (a–j) Fluoromicrographic images of the MDA-MB-231 cells 24 h after treatment with various conditions of I_PE_C_CN_AHG. X1 and X2 denote the cells treated with AHG and AHG + NIR, respectively, followed by maintenance at 37 °C for 24 h. The green spots represent live cells stained with calcein-AM. Scale bar: 1 mm.

The phototoxicity of I_PE_C_CN_AHG to TNBC cells was first examined by using I_PE_AHG as the test material through which the chemotoxicity could be completely excluded. As shown in Figure 4A, >90% of the cells survived after treatment with NIR in the absence of ICG, indicating that the NIR-induced slight temperature elevation (Figure 2C) is nontoxic. A dose-dependent cytotoxicity can be found in all the ICG-treated groups, where I_PE_AHG + NIR dramatically reduced cell viability from 95 to 42% as the concentration of ICG was increased from 0 (AHG) to 160 μM. These outcomes show that the toxicity of AHG was negligible, while IPNEs-loaded AHG was indeed able to destroy cancer cells upon NIR irradiation. Free ICG + NIR conferred the highest cell mortality among all of the treatment regimens throughout the dose range. However, naked ICG is not a suitable material for practical use because it is photo- and thermally susceptible and easy to remove from the circulation that is unfavorable for use in vivo.^17,18^

We subsequently investigated the photochemotoxicity of I_PE_C_CN_AHG with [ICG] = 80 or 160 μM incorporation with various CPT dosages to TNBC cells. Figure 4B shows the in situ conditions of the cells treated by AHG ± NIR or I_PE_C_CN_AHG + NIR with different dosage settings 24 h after the experiment. Based on MTT analysis as plotted in Figure 4C, the viability of cells treated with I_PE_C_CN_AHG + NIR was significantly lower than that with an equal dose of free CPT throughout the CPT dose range (*P* < 0.05 for all). I_PE_C_CN_AHG + NIR with [CPT] ≥ 300 μM can provide a significantly enhanced cancericidal effect compared to I_PE_AHG + NIR with equal ICG dosage (*P* < 0.05). Moreover, the cell viability with I_PE_C_CN_AHG + NIR was even lower than that caused by using a double amount of encapsulated CPT alone. These results demonstrate that phototherapy indeed plays a crucial role in I_PE_C_CN_AHG-mediated anticancer treatment. With the advantages of enhanced ICG stability, increased singlet oxygen yields, and effective cancericidal functionality in vitro, I_PE_C_CN_AHG proceeded to an animal model for in vivo efficacy investigations.

### 3.8 Tumoricidal Effects of I_PE_C_CN_AHG In Vivo

**Figure 5:**
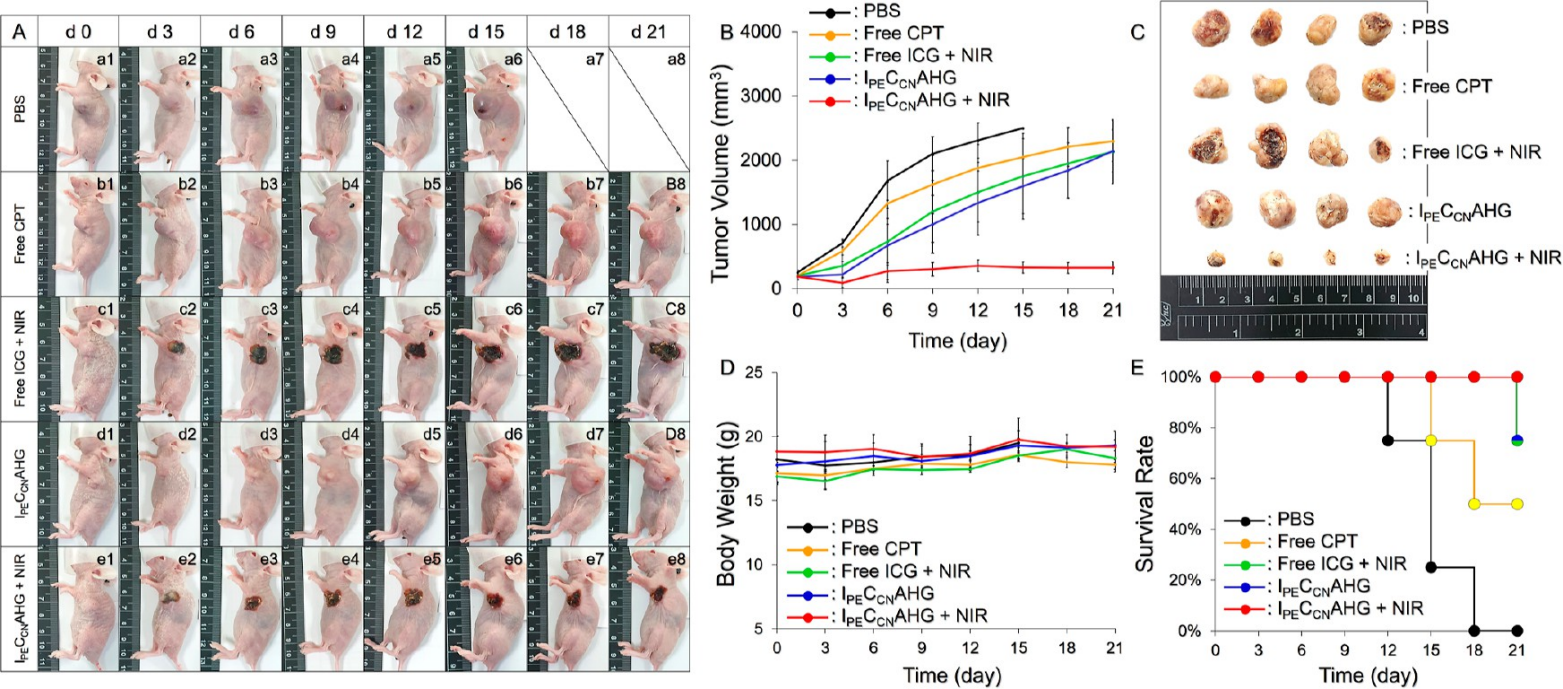
In vivo antitumor efficacy of I_PE_C_CN_AHG. (A) Photographs of the tumor-bearing mice showing different conditions of tumor growth within the 21 day treatment. The mice in the PBS group were all sacrificed early in the first 15 days owing to oversized tumors. (B) Variations of tumor size of all groups under different treatments for 21 days. (C) Photograph of all tumors collected after sacrifice. (D,E) Variations of body weight (D) and survival rate (E) of the mice under different treatments for 21 days. Values in (B,D) are the mean ± SD (*n* = 4).

To provide sufficient photo- and chemotherapeutics for cancer treatment, I_PE_C_CN_AHG with 160 μM ICG and 300 μM CPT was selected in the animal assay. Figure 5A shows the tumor growth conditions of the mice that received different treatments within 21 days, in which the subjects with PBS were all absent beyond the 18th day (Figure 5A, a7 and a8) because they were sacrificed early due to oversized tumors (*V* ≥ 2000 mm^3^). Based on the analysis of the tumor size plotted in Figure 5B, free CPT (Figure 5A, row b) and I_PE_C_CN_AHG without NIR (Figure 5A, row d) were not able to suppress the growth of cancer cells, as the tumor sizes were dramatically enhanced by 11.5- and 11.9-fold, respectively, after 21 days. Free ICG + NIR destroyed the tumors that were exposed to NIR (Figure 5A, row c), while those without light exposure continuously proliferated and showed a 10.7-fold enlarged size after 21 days. Only I_PE_C_CN_AHG + NIR can successfully inhibit the growth of cancer cells, and the mean size of the tumor was barely augmented by 74.2% after 21 days. All tumors were collected and photographed after sacrifice, as presented in Figure 5C. Furthermore, neither tumor recurrence (Figure 5B) nor significant weight loss (Figure 5D) was observed in the mice with I_PE_C_CN_AHG + NIR during treatment, and a 100% survival rate was obtained for the group after 21 days (Figure 5E). These outcomes indicate that the I_PE_C_CN_AHG with 160/300 μM of ICG/CPT in association with 60 s NIR irradiation (808 nm; 6 W/cm^2^) was efficacious in arresting the growth of TNBC tumors in vivo.

Based on the detection of tumor growth shown in Figure 5B, I_PE_C_CN_AHG without NIR exhibited a higher antitumor efficacy than free CPT during the 21 day treatment. Such results could be explained by the fact that CPT in I_PE_C_CN_AHG was protected by both polymeric carriers and the hydrogel matrix and thus had a longer retention time in tumors than free agents. Furthermore, CPT at a concentration of 300 μM can barely kill <30% of MDA-MB-231 cells in vitro as illustrated in Figure 4B, showing that such a dose alone is not enough to destroy tumors in vivo. However, CPT is critical and indispensable in the tumoricidal process, since it can provide sustained antitumor activity after phototherapy to ensure successful anticancer effects. Otherwise, the surviving cells after NIR irradiation may grow and therefore fail the treatment as occurred in the group with free ICG + NIR (Figure 5B). Given that the I_PE_C_CN_AHG was equipped with both photo- and chemotherapeutic functionalities, the tumors in this group could be successfully arrested upon NIR irradiation. Furthermore, considering that only <10% of free CPT from I_PE_C_CN_AHG could be detected after NIR irradiation (Figure 2E), we reason that such anticancer efficacy was accomplished by phototherapy followed by CCNPs-mediated chemotherapy, whereby cancer cells were first destroyed by IPNEs-derived hyperthermia and singlet oxygen, followed by sustained damage with CPT after cellular internalization of CCNPs, a two-stage tumoricidal process. We surmise that the released CCNPs can be quickly adhered to and engulfed by surviving cancer cells due to electrostatic attractions between the cells and CCNPs which carry positive charges, as shown in Figure 1E. Moreover, I_PE_C_CN_AHG is predicted to provide not only effective anticancer therapy but also reduced multidrug resistance and/or chemotoxicity due to the use of less CPT (<IC_50_).

### 3.9 Prognosis of TNBC after I_PE_C_CN_AHG-Mediated Photochemotherapy

**Figure 6:**
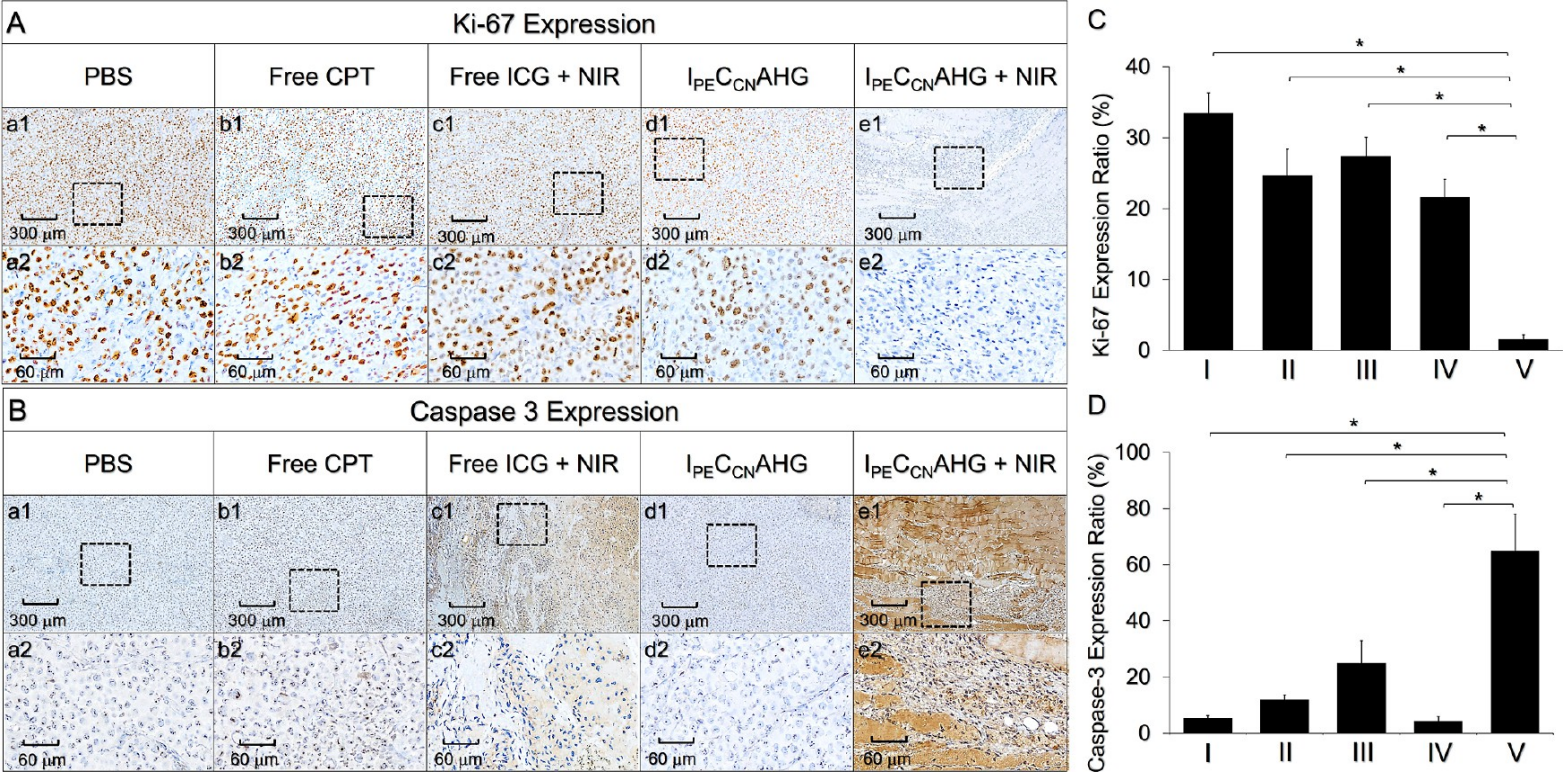
Histological analysis of the tumors after various treatments. (A,B) *K*_i_-67 (A) and caspase-3 (B) IHC staining images of the tumors under various treatments for 21 days. (a2–e2) are the magnified images of the area framed in (a1–e1). (C,D) Expression levels of *K*_i_-67 (C) and caspase-3 (D) of the tumors after different treatments for 21 days. The expression percentage was obtained by calculating the staining ratio of the marker over the whole area using ImageJ software. I, II, III, IV, and V represent the groups treated with PBS, free CPT, free ICG + NIR, I_PE_C_CN_AHG, and I_PE_C_CN_AHG + NIR, respectively. Values are the mean ± SD (*n* = 4). *P < 0.05.

All tumors were histologically analyzed by *K*_i_-67 and caspase-3 IHC staining assays immediately after collection. The *K*_i_-67 protein, also known as MKI67, has long been recognized as a marker for cellular proliferation.^35^ It is expressed in the active cells (G1, S, G2, and M phases) but is absent in the resting ones (G0 phase),^36^ making it a biomarker for diagnosis and prognosis of breast cancer including TNBC.^37−39^ On the other hand, the caspase cascade is an ensemble of critical signaling molecules involved in cell apoptosis, in which caspase-3 is one of the most commonly used effectors since it plays a critical role in both receptor and mitochondrial pathways of cell death.^40^ Moreover, caspase-3 activation is needed for induction of apoptosis in response to a variety of chemo-drugs including CPT.^41^ In this study, the group with I_PE_C_CN_AHG + NIR (Figure 6A, column e) was found to show a relatively mild *K*_i_-67 but strong caspase-3 expression (Figure 6B, column e) compared to the other four settings (Figure 6A,B, column a–d), indicating that the tumor cells progressed predominantly toward death instead of proliferation. The differences between the IHC staining results for each biomarker were further confirmed through quantitative analyses of their expression levels, as plotted in Figure 6C,D. These outcomes suggest that the incidence of tumor recurrence in the group with I_PE_C_CN_AHG + NIR was relatively low, and such effectiveness of tumor suppression can also be verified by its smaller tumor size as presented in Figure 5A–C.

### 3.10 Systematic Toxicity of I_PE_C_CN_AHG

**Figure 7:**
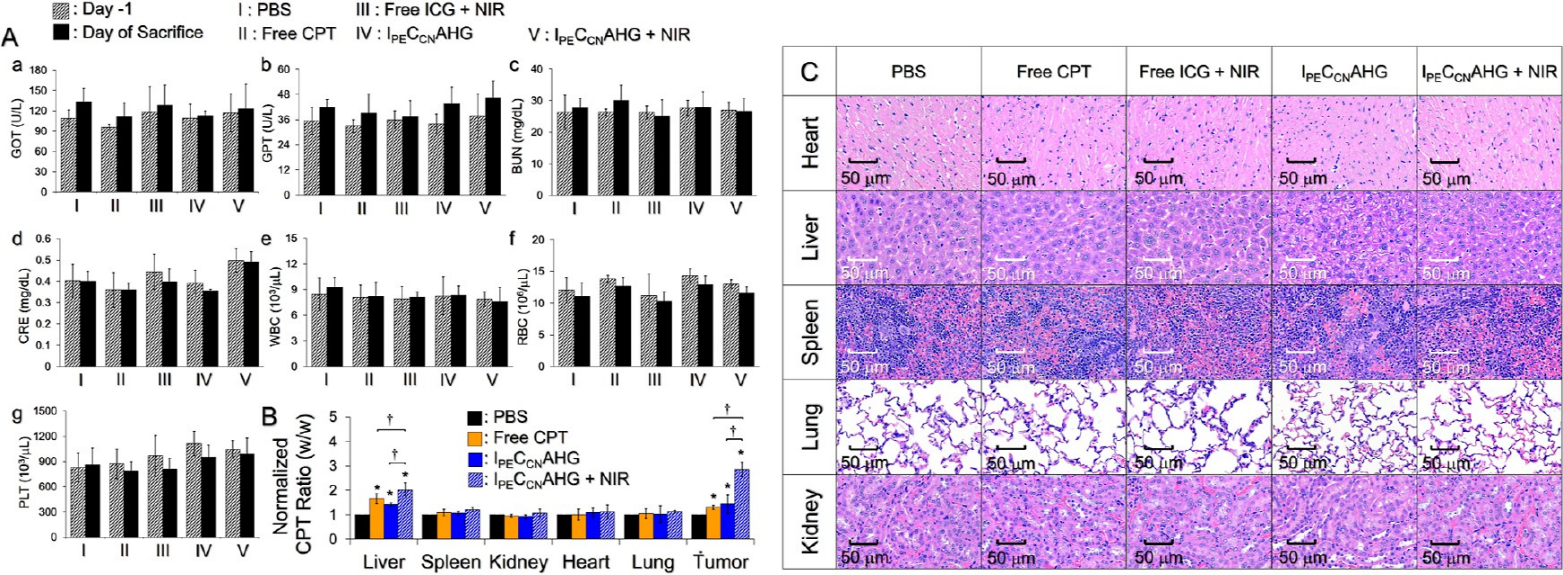
Analysis of systemic toxicity of I_PE_C_CN_AHG. (A) Expression levels of the serum markers regarding liver (a,b) and kidney (c,d) functions, as well as the numbers of white blood cells (WBCs/e), red blood cells (RBCs/f), and platelets (PLTs/g) of the mice, which were measured 24 h before treatment (day-1) and right before sacrifice. Values are the mean ± SD (*n* = 4). (B) Analysis of the quantity of CPT remaining in the five organs and tumors of the mice after different treatments for 21 days. Values are the mean ± SD (*n* = 4). *P < 0.05 compared to the value gained from the PBS group in the same organ set. ^†^*P* < 0.05. (C) Photomicrographs of H&E staining of the five organ tissues after various treatments for 21 days.

Figure 7A shows the results of biochemical analyses of blood for all tumor-bearing mice before and after treatment. Our data show that the values in the drug-treated groups were all similar to those in the PBS group at the same time points (*P* = NS for all), indicating that the effects of I_PE_C_CN_AHG + NIR on the liver (Figure 7A, a,b), kidney (Figure 7A, c,d), and blood cells (Figure 7A, e–g) of the experimental mice were negligible within the 21 day treatment.

The conditions of CPT remaining in vivo for the groups with free CPT, I_PE_C_CN_AHG, and I_PE_C_CN_AHG + NIR were additionally investigated after sacrifice. As shown in Figure 7B, a significantly higher amount of CPT can be found in the tumor and liver of the group with I_PE_C_CN_AHG + NIR (*P* < 0.05 for each). Except for the above two organs where the agents were directly administered and metabolized,^42^ the three modalities gave comparable CPT accumulation in the other four tissues, and those values were all similar to that obtained from the PBS group (*P* = NS for each). These results can be explained by the fact that naked CPT was quickly removed by physiological cleanup mechanisms such as transcapillary filtration and the reticuloendothelial system, while CPT in the I_PE_C_CN_AHG was highly stabilized by dual polymeric carriers (i.e., AHG and CNPs) that dramatically reduced its release efficiency to the system (Figure 2D). In terms of the group with I_PE_C_CN_AHG + NIR, we reason that quite a few CCNPs were discharged from the gel upon NIR irradiation, where the entrapped CPT can be protected from external enzymatic attacks but is able to be released inside cells due to the biodegradability of chitosan,^43,44^ thereby leading to a significant cumulative amount in vivo. Nevertheless, neither significant lesion nor inflammation was found in organs with I_PE_C_CN_AHG + NIR as compared to the conditions of the PBS group shown in Figure 7C. Taken together, these results clearly demonstrate the bioavailability of I_PE_C_CN_AHG + NIR for medical applications.

## 4 Conclusions

In summary, an injectable alginate complex hydrogel loaded with IPNEs and CCNPs, named I_PE_C_CN_AHG, was developed for the photochemotherapy of TNBC. IPNEs with PFOB can induce hyperthermia and generate more singlet oxygen than an equal dose of free ICG upon NIR irradiation to achieve photothermal and photodynamic therapy. CCNPs with positive charges may facilitate cell internalization and provide sustained release of CPT to carry out chemotherapy. I_PE_C_CN_AHG integrating IPNEs and CCNPs enabled stage-wise combinational therapeutics that may overcome the issues of detrimental side effects and multidrug resistance occurring in most chemotherapy. Through the animal assay, we demonstrated that the growth of TNBC tumors can be significantly arrested by the I_PE_C_CN_AHG with 160/300 μM of ICG/CPT in association with 60 s of NIR irradiation (808 nm; 6 W/cm^2^) without having notable systemic toxicity in vivo. We reason that such tumor-inhibitory efficacy was accomplished by phototherapy, followed by chemotherapy, a two-stage antitumor process. Given the above anticancer efficacies together with its advantage of biocompatibility, the developed I_PE_C_CN_AHG is anticipated to be a feasible tool for use in clinical TNBC treatment.
